# Newer indications for neuromonitoring in critically ill neonates

**DOI:** 10.3389/fped.2023.1111347

**Published:** 2023-04-28

**Authors:** Gabriel F. T. Variane, Rafaela F. R. Pietrobom, Caroline Y. Noh, Krisa P. Van Meurs, Valerie Y. Chock

**Affiliations:** ^1^ Division of Neonatology, Department of Pediatrics, Irmandade da Santa Casa de Misericórdia de São Paulo, São Paulo, Brazil; ^2^ Clinical Research Department, Protecting Brains and Saving Futures Organization, São Paulo, Brazil; ^3^ Division of Neonatal and Developmental Medicine, Stanford University School of Medicine and Packard Children's Hospital Stanford, Palo Alto, CA, United States

**Keywords:** neuromonitoring, near-infrared spectroscopy, amplitude-integrated electroencephalography, multimodal monitoring, brain injury, neurocritical care, cerebral function

## Abstract

Continuous neuromonitoring in the neonatal intensive care unit allows for bedside assessment of brain oxygenation and perfusion as well as cerebral function and seizure identification. Near-infrared spectroscopy (NIRS) reflects the balance between oxygen delivery and consumption, and use of multisite monitoring of regional oxygenation provides organ-specific assessment of perfusion. With understanding of the underlying principles of NIRS as well as the physiologic factors which impact oxygenation and perfusion of the brain, kidneys and bowel, changes in neonatal physiology can be more easily recognized by bedside providers, allowing for appropriate, targeted interventions. Amplitude-integrated electroencephalography (aEEG) allows continuous bedside evaluation of cerebral background activity patterns indicative of the level of cerebral function as well as identification of seizure activity. Normal background patterns are reassuring while abnormal background patterns indicate abnormal brain function. Combining brain monitoring information together with continuous vital sign monitoring (blood pressure, pulse oximetry, heart rate and temperature) at the bedside may be described as multi-modality monitoring and facilitates understanding of physiology. We describe 10 cases in critically ill neonates that demonstrate how comprehensive multimodal monitoring provided greater recognition of the hemodynamic status and its impact on cerebral oxygenation and cerebral function thereby informing treatment decisions. We anticipate that there are numerous other uses of NIRS as well as NIRS in conjunction with aEEG which are yet to be reported.

## Introduction

Noninvasive neuromonitoring allows continuous screening and assessment of neurologic status at the bedside. With growing attention to the impact of brain injury on long-term neurodevelopmental outcomes, neuromonitoring has gained more attention as an essential element of neurocritical care in the neonatal intensive care unit (NICU) (1–3). Neurologic monitoring with near-infrared spectroscopy (NIRS), amplitude-integrated electroencephalography (aEEG), or continuous video EEG (cEEG) has been commonly employed in the care of infants at high risk for brain injury and neurologic sequelae including extremely preterm neonates and those with intraventricular hemorrhage or hypoxic-ischemic encephalopathy (4, 5). However, there are other conditions for which multimodal neuromonitoring has proven to be helpful in diagnosing pathologic conditions and guiding clinical care in the NICU (6).

NIRS is a noninvasive technology for continuous bedside monitoring of regional tissue hemoglobin oxygen saturation. NIRS values, or rSO_2_, reflect the balance between oxygen delivery and consumption at any cerebral or somatic site by reading a venous-weighted oxygen saturation (1). In comparison, peripheral oxygen saturation, or SpO_2_, and arterial oxygen saturation, or SaO_2_, reflect the level of oxygen being delivered from the arterial blood. Multisite monitoring of regional oxygenation provides organ-specific assessment providing data on the differing impact of changes in perfusion on various organs and their response to interventions. Cerebral oxygen saturation (rScO_2_) is most commonly monitored as the brain is a highly metabolic organ with tight autoregulatory mechanisms to maintain constant blood flow over a wide range of arterial pressures (2, 3). As renal perfusion and oxygenation are more dependent on the cardiac output, a change in renal oxygen saturation (rSrO_2_) is often the earliest indicator of alterations in systemic perfusion and oxygenation. Cardiac failure, low cardiac output, or decreased blood volume may be detected by a decrease in rSrO_2_ before any other clinical parameters are apparent (4). On the other hand, hypercarbia, even in the absence of changes in blood pressure (BP) or arterial oxygen saturation, is known to cause cerebral vasodilation and increase cerebral blood flow. Thus, rScO_2_ may be affected when rSrO_2_ remains constant. Other factors influencing rScO_2_ include anemia, hypoglycemia, intraventricular hemorrhage, and posthemorrhagic ventricular dilatation (1). In addition, recent studies have indicated that mesenteric saturation (rSmO_2_) is useful in assessing gut oxygenation in neonates with bowel pathology, despite the frequent variability typical of this site (7). rSmO_2_ provides additional data that may correlate with clinical parameters and identify necrotizing enterocolitis (NEC), bowel ischemia, and other pathologies thus assisting in determining the optimal timing for a surgical intervention (7).

Amplitude-integrated electroencephalography (aEEG) has been increasingly used in neonatal intensive care units (NICU) and is most well-known for its use in babies with hypoxic-ischemic encephalopathy (5). aEEG monitors display both the raw EEG and compressed aEEG tracing from the bihemispheric channels in the central and parietal regions and conveniently allows continuous bedside evaluation of cerebral background activity patterns and thus the level of cerebral function as well as allowing for identification of seizure activity. While the use of NIRS and aEEG has been evaluated and established separately, limited literature reports the advantages and added value of their simultaneous use in the NICU (6).

In this manuscript, we describe 10 cases in critically ill neonates including cardiopulmonary disorders, circulatory disorders and abdominal disorders, in which comprehensive multimodal monitoring with multisite NIRS with and without simultaneous use of aEEG provided greater knowledge of the hemodynamic status and its impact on cerebral function and injury. These cases were obtained from Neuro-NICUs located at level III and IV NICUs in Brazil and the U.S., where NIRS and aEEG are routinely utilized in specific populations of neonates at risk for brain injury (8). The INVOS 5100C Cerebral/Somatic Oximeter (Medtronic, Minneapolis, MN) with neonatal sensors were used for NIRS monitoring; the CFM Olympic Brainz Monitor (Natus, Pleasanton, CA) and Neuron-Spectrum 4 (Neurosoft, Ivanovo, Russia) were used for aEEG/EEG monitoring.

## Cardiopulmonary disorders

### Case 1—pneumothorax

#### Case summary

**Figure 1:**
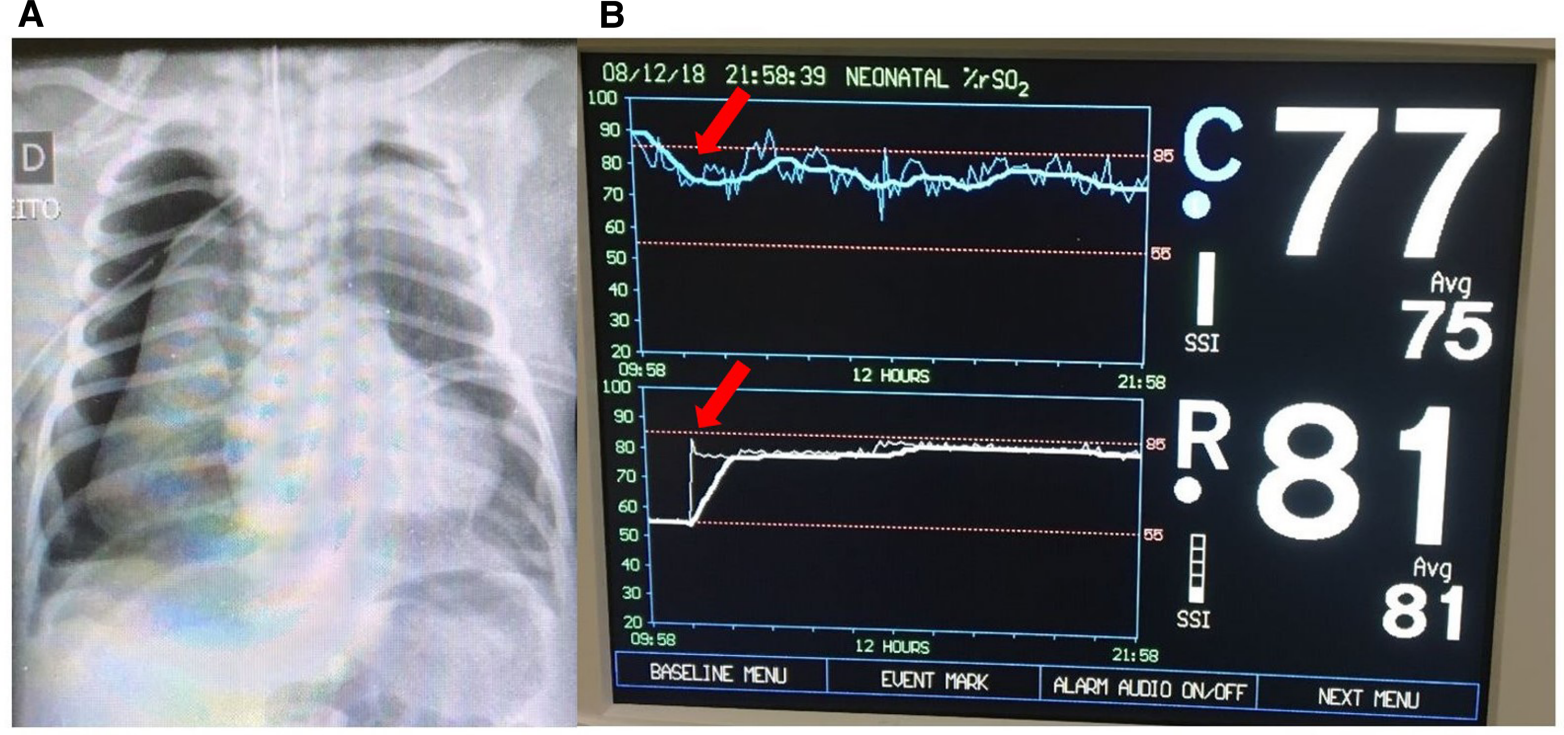
(**A**) Chest x-ray showing bilateral pneumothoraces. (**B**) Improvement of NIRS findings immediately after pneumothorax treatment with needle aspiration (arrows). The rScO_2_ (blue trend line) decreased to normal levels (∼75% to 77%) and rSrO_2_ (white trend line) increased to 81%.

A late preterm infant was admitted to the NICU at 24 h of life due to respiratory distress and cyanosis requiring intubation. The infant experienced a sudden decompensation with a drop in mean blood pressure (MBP) to 32 mmHg and pulse oximetry to 75%. Measures to increase the SpO_2_ were implemented immediately, including increase of FiO_2_ to 100%. NIRS monitoring was then initiated and showed rScO_2_ ∼90% and rSrO_2_ ∼55%. The closest recorded SpO_2_ was 91%. Blood gas analysis showed PaCO_2_ 99 mmHg, and the chest radiograph demonstrated bilateral pneumothorax (Figure 1A). Needle aspiration and chest drainage were performed with clinical improvement (mean BP 40 mmHg and SpO_2_ 92%). The rScO_2_ decreased from 90% to 75%–77%, accompanied by a substantial increase in rSrO_2_ to 81% (Figure 1B). The repeat blood gas showed a reduction in PaCO_2_ to 56 mmHg.

#### Physiology

Tension pneumothorax leads to compression of the mediastinum, decreased systemic venous return, and consequently results in shock. Hemodynamic and ventilatory instability in the neonatal period may result in altered cerebral saturation, even in the presence of normal pulse oximetry. Hypercapnia leads to cerebral vasodilation and a shift of the oxyhemoglobin dissociation curve to the right, reducing hemoglobin oxygen affinity (9, 10). Ongoing assessment of rScO_2_ patterns has been shown to provide information on hypercapnia-induced cerebral hyperperfusion. Dix et al. (11) studied a cohort of 38 infants with 60 detected episodes of hypercapnia using continuous endotracheal (ET) CO_2_ measurement. An association between high ET CO_2_ and supranormal rScO_2_ values was noted. In case 1, the hemodynamic consequences due to pneumothorax may explain the initial lower rSrO_2_ while the high cerebral saturations may be related to very high pCO_2_ values causing cerebral vasodilation. After treatment of the pneumothorax, with normalization of blood pressure and correction of hypercapnia, rScO_2_ values decreased and rScO_2_ increased. Identifying abnormal rScO_2_ trends during hypercapnia may lead to early diagnosis and treatment of pneumothorax in mechanically ventilated infants, resulting in the normalization of brain perfusion and oxygenation. Dual site NIRS monitoring assists in hemodynamic evaluation, together with other parameters such as urine output, capillary refill, and blood pressure. Somatic desaturation is commonly an early indicator of shock and may persist until hemodynamic stability is restored (12, 13).

### Case 2—hypotension and bradycardia

#### Case summary

**Figure 2:**
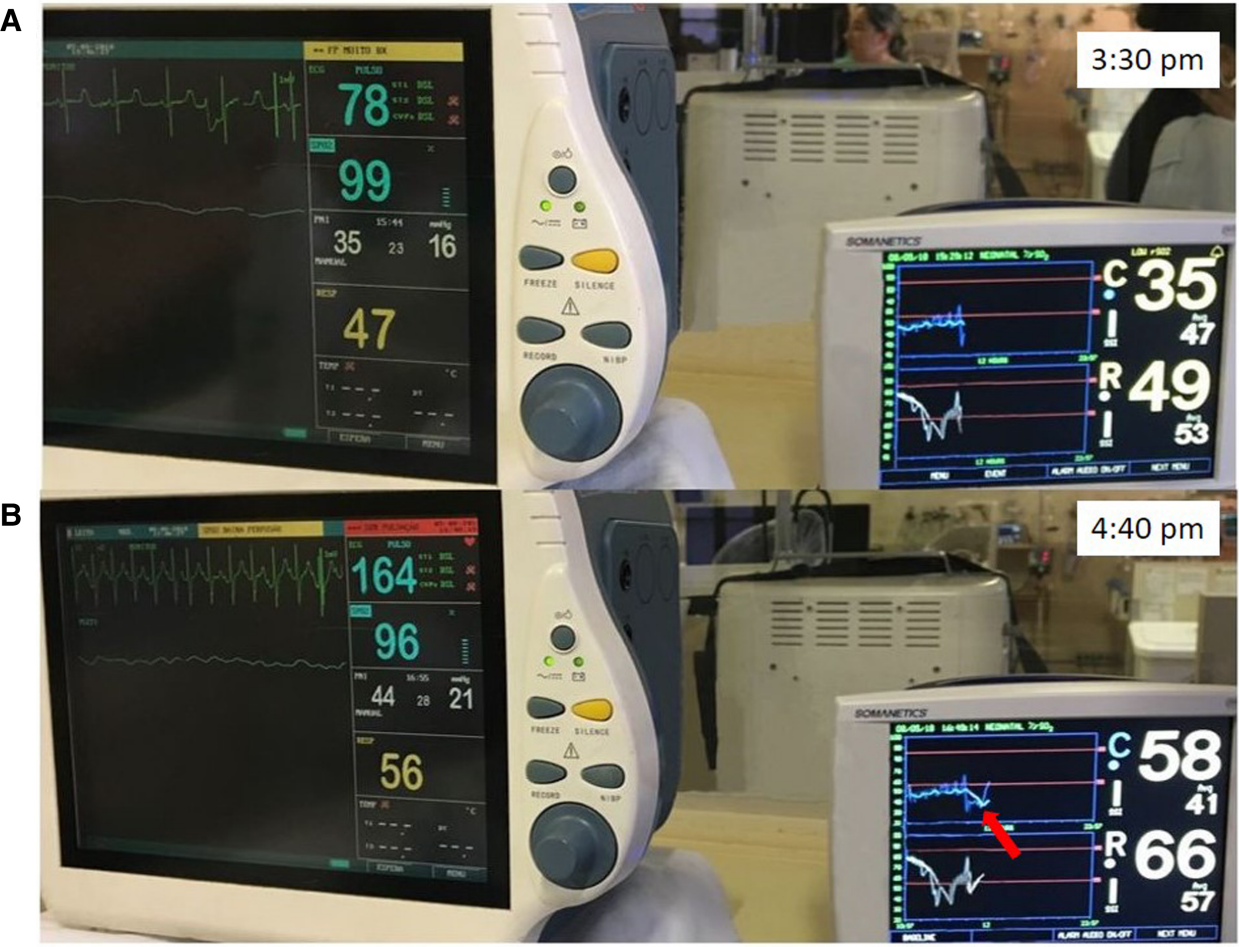
(**A**) Bradycardia due to hyperkalemia with associated hemodynamic compromise and low rScO_2_ (blue trend line) and rSrO_2_ (white trend line) values. (**B**) After improvement of heart rate due to treatment, an improvement in rScO_2_ to ∼58% (arrow) and rSrO_2_ to ∼60% to 66% was noted.

An extremely preterm infant less than 24 weeks of gestation required intubation in the delivery room. The infant was admitted to the NICU and started on empiric antibiotic therapy. The initial blood culture was positive for Escherichia coli. On the second day of life, the infant was oliguric with bradycardia to 78bpm, hypotensive with a mean BP of 23 mmHg and had delayed capillary refill despite normal SpO_2_. NIRS monitoring was initiated demonstrating rScO_2_ ∼35% to 45% and rSrO_2_ ∼40% to 50% (Figure 2A). Renal failure was diagnosed with hyperkalemia (K = 10 mEq/l). Calcium gluconate infusion was administered, together with other measures to reduce serum potassium. After treatment, there was an improvement in heart rate to a normal range for age associated with an increase in rScO_2_ to ∼58% and rSrO_2_ to ∼60% to 66% (Figure 2B).

#### Physiology

Clinical parameters are not always sufficient for hemodynamic evaluation of preterm infants and the evaluation of new non-invasive techniques to detect hemodynamic impairment is important. Previous studies have shown that low blood pressure and bradycardia, independent of hypoxemia, commonly affects regional tissue oxygenation (14). In case 2, there was pronounced reduction in both rScO_2_ and rSrO_2_ values. These findings may be explained by immature cerebral autoregulation in the extremely preterm and critically ill infant. The precise period when cerebral autoregulation becomes mature is unknown (15). Walter et al. (16) studied a cohort of two groups of neonates at 26–31 weeks' (very preterm) and 32–38 weeks' (late preterm) gestation with bradycardia episodes. Moderate and severe bradycardia were associated with decreased brain oxygenation in both groups. The impact of bradycardia was more severe in very preterm newborns when compared to late preterm infants. Tissue oxygen nadirs below 55% were found in 61% of moderate-severe bradycardic events and 35% of mild bradycardic events in the very preterm population. Even minor bradycardias were associated with reductions in brain oxygenation. These findings highlight the relevance of assessing cerebral oxygenation in preterm newborns with significant bradycardia.

### Case 3—pericardial effusion

#### Case summary

**Figure 3:**
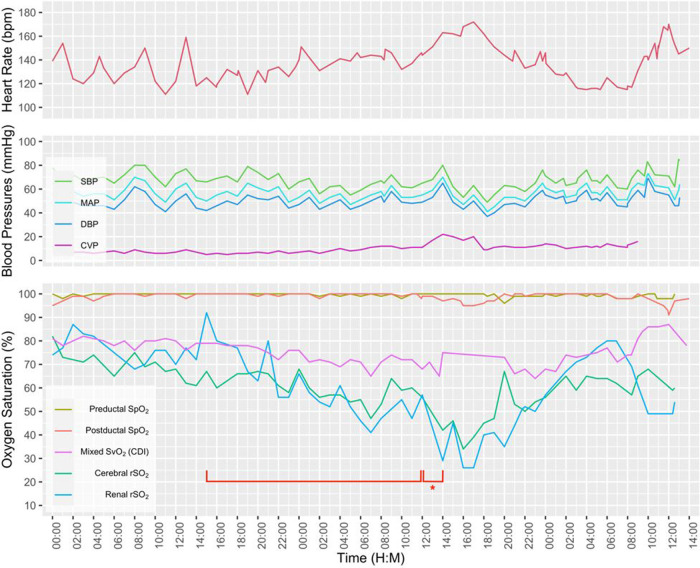
A multimodal graphic including vital signs and hemodynamic parameters. Note the gradual decline in renal and cerebral rSO_2_ over a 21-hour period (bracket), followed by a 2-hour period with abrupt decrement (bracket with an asterisk).

A term neonate with hypoxic respiratory failure due to meconium aspiration syndrome and persistent pulmonary hypertension was placed on venoarterial extracorporeal life support (ECLS) on the day of life 3 due to persistent hypoxemia. An echocardiogram showed normal anatomy and suprasystemic right ventricular pressures. A chest radiograph demonstrated the arterial cannula tip at T3 and venous cannula tip at T10. After being on full ECLS support for 7 days, pump flow was gradually weaned due to improved lung expansion. While pump flow was weaned from 75 to 20 ml/kg/min, systemic oxygenation and perfusion as measured by SpO_2_, arterial blood gases, blood pressures, and urine output remained stable. However, both rScO_2_ and rSrO_2_ steadily decreased over a 21-hour period followed by a 2-hour period with an abrupt decrement (Figure 3). A point of care ultrasound was performed, and a large pericardial effusion was seen. The tip of the venous catheter was located at the inferior cavoatrial junction, which was confirmed by an echocardiogram. A pericardial drain was placed with the removal of 49 ml of bloody fluid while on full ECLS. There was subsequent improvement in both rScO_2_ and rSrO_2_. The decannulation was performed on ECLS day 8 and the pericardial drain was removed 48 h following decannulation. The infant was discharged in stable condition with a normal respiratory and neurologic examination.

#### Physiology

NIRS is being used more frequently in ECLS patients due to their critical illness. In this setting, the primary focus is often on monitoring brain oxygenation. This case demonstrates the value of two site monitoring and how it provided an understanding of changes in perfusion to both the brain and kidneys. Despite a large pericardial effusion, there was no evidence of clinical instability based on vital signs and ECLS parameters. Monitoring of cerebral and renal NIRS easily detected the hemodynamic effect of the pericardial effusion, demonstrating a gradual decrease in perfusion to the cerebral and somatic tissue beds.

### Case 4—congenital diaphragmatic hernia (CDH)

#### Case summary

**Figure 4:**
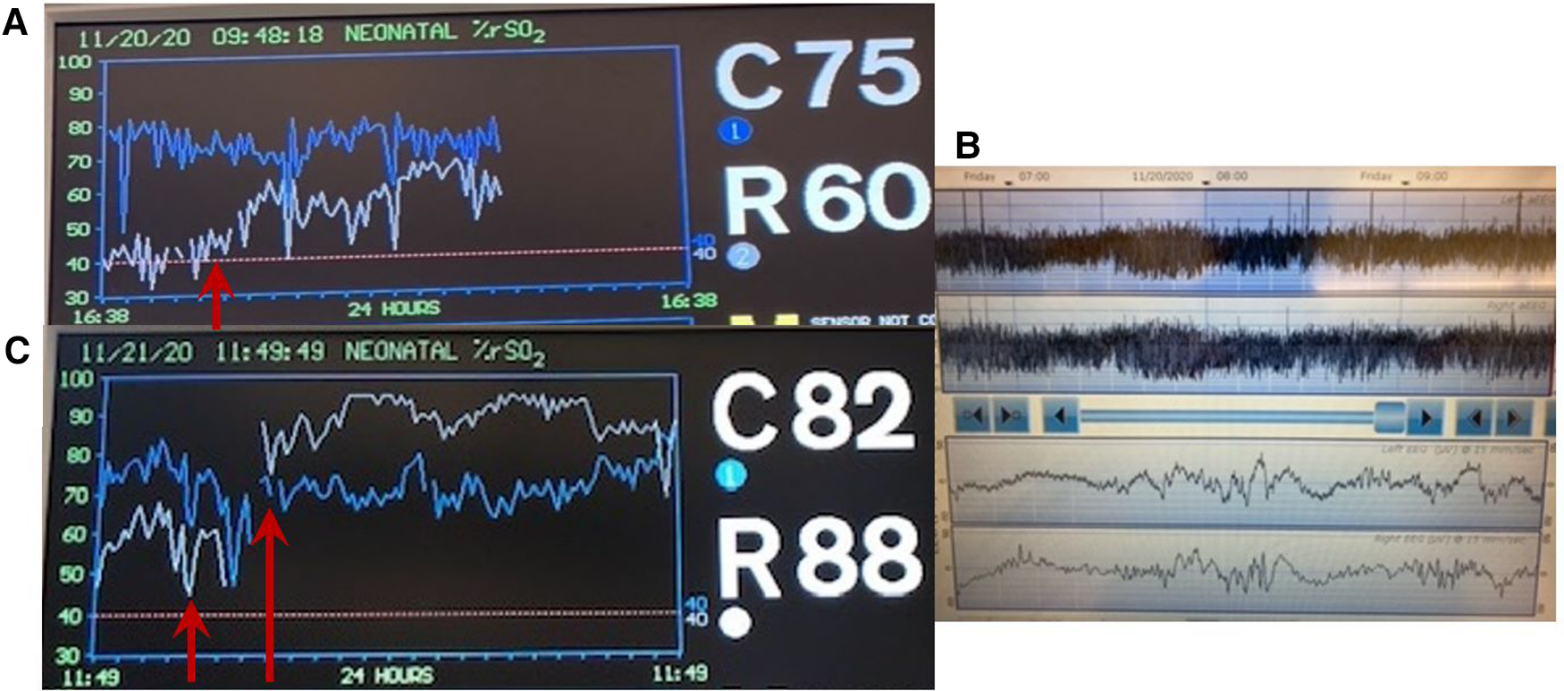
(**A**) Increase in rSrO_2_ (white trend line) is seen after initiation of pressor support (arrow). (**B**) Reassuring aEEG tracing performed at the same time as (**A**) demonstrates continuous normal voltage tracing with sleep wake cycling. (**C**) A decrease in both rSrO_2_ (white trend line) and rScO_2_ (blue trend line) occurred prior to decision to initiate ECLS when pre-ductal SpO_2_ was 70% (first arrow). Subsequent improvement in NIRS measures is then seen (second arrow).

A term infant with a prenatal diagnosis of left-sided congenital diaphragmatic hernia (CDH) was born by scheduled Cesarean section delivery. Fetal magnetic resonance imaging (MRI) showed half of the liver, stomach, and bowel in the chest with estimated lung volumes <25% of expected. The infant was intubated in the delivery room and despite a gentle ventilation and permissive hypercapnia approach, clinical support escalated quickly to include high frequency oscillatory ventilation with FiO_2_ 100% and inhaled nitric oxide 20 ppm. Pre-ductal SpO_2_ was 90%–100% and post-ductal SpO_2_ 20%–30%. Lactate was <2 and there was no evidence of acidosis. Echocardiogram demonstrated a dilated right ventricle (RV) with elevated RV pressures, right-to-left shunting across the patent ductus arteriosus, and mildly decreased function. Neuromonitoring was initiated in this critically ill infant with respiratory failure. rScO_2_ was 70%–80% and rSrO_2_ was 30%–45%. After initiation of pressor support to improve blood pressure, the rSrO_2_ increased to 55%–65% (Figure 4A). Simultaneous aEEG monitoring demonstrated continuous normal voltage with sleep-wake cycling (Figure 4B). Given the reassuring neuromonitoring and evidence of adequate end organ perfusion, the clinical support was continued without any changes. However, after 24 h, both rScO_2_ and rSrO_2_ decreased to 45% and 43%, respectively. These findings coincided with a decrease in preductal SpO_2_ to 70%. There was no change in the aEEG background pattern. Lactate increased to 3.1 mg/dl and post-ductal arterial blood gas showed pH 7.31 PaCO_2_ 59 PaO_2_ 27 HCO_3_ 30 Base excess 3. Given these changes, the decision was made to initiate ECLS. Subsequently, both cerebral and renal perfusion significantly improved to 80% and 90% respectively (Figure 4C).

#### Physiology

CDH is a condition associated with significant risk for respiratory failure, pulmonary hypertension, and possible need for ECLS. Neuromonitoring may be particularly beneficial in this situation to guide clinical management and confirm the decision for or against initiation of ECLS. In the case described above, appropriate end organ saturations (both brain and kidney) combined with reassuring brain function as measured by aEEG aided the initial clinical decision to avoid ECLS. However, a later decline in both rScO_2_ and rSrO_2_ preceded an eventual decline in preductal SpO_2_ measures and acidosis, leading to the decision to place the infant on ECLS. As aEEG changes are typically also preceded by abnormal NIRS values (6), initiation of ECLS was performed even before aEEG changes could occur. While dramatic improvement in NIRS values occurred after initiation of ECLS, in the context of impaired cerebral autoregulation, there remains a risk for hemorrhage with sudden increases in cerebral perfusion. Continued neuromonitoring is warranted during the ECLS period.

Prenatal and postnatal predictors of ECLS have been extensively studied with variable utility, although investigation of tissue oxygenation has been limited in this population. In a cohort of 56 neonates with CDH, a difference between right and left hemispheric cerebral oxygenation of >10% in the first 12 h of life was predictive of ECLS (AUC 0.92, *p* < 0.001) with a sensitivity of 85% and specificity of 100% (17). Moreover, right hemispheric rScO_2_ was lower in those eventually requiring ECLS and values normalized after cannulation. It has been speculated that pulmonary hypertension, low left ventricular output, and retrograde ductal perfusion may lead to differential perfusion of the left and right carotid arteries and subsequent left-right rScO_2_ differences (17).

Low renal saturation in the CDH population may also be useful in guiding clinical interventions. As renal perfusion is highly dependent on cardiac output, a low rSrO_2_ may be the first indication of declining systemic perfusion before signs of oliguria or acidosis are noted. As in this scenario, it may prompt initiation of pressor support. In a small study of 6 neonates with CDH on ECLS, lower renal NIRS measures preceded a decline in mean arterial blood pressure and correlated with decreased urine output (18).

## Circulatory disorders

### Case 5—persistent pulmonary hypertension of the newborn (PPHN)

#### Case summary

**Figure 5:**
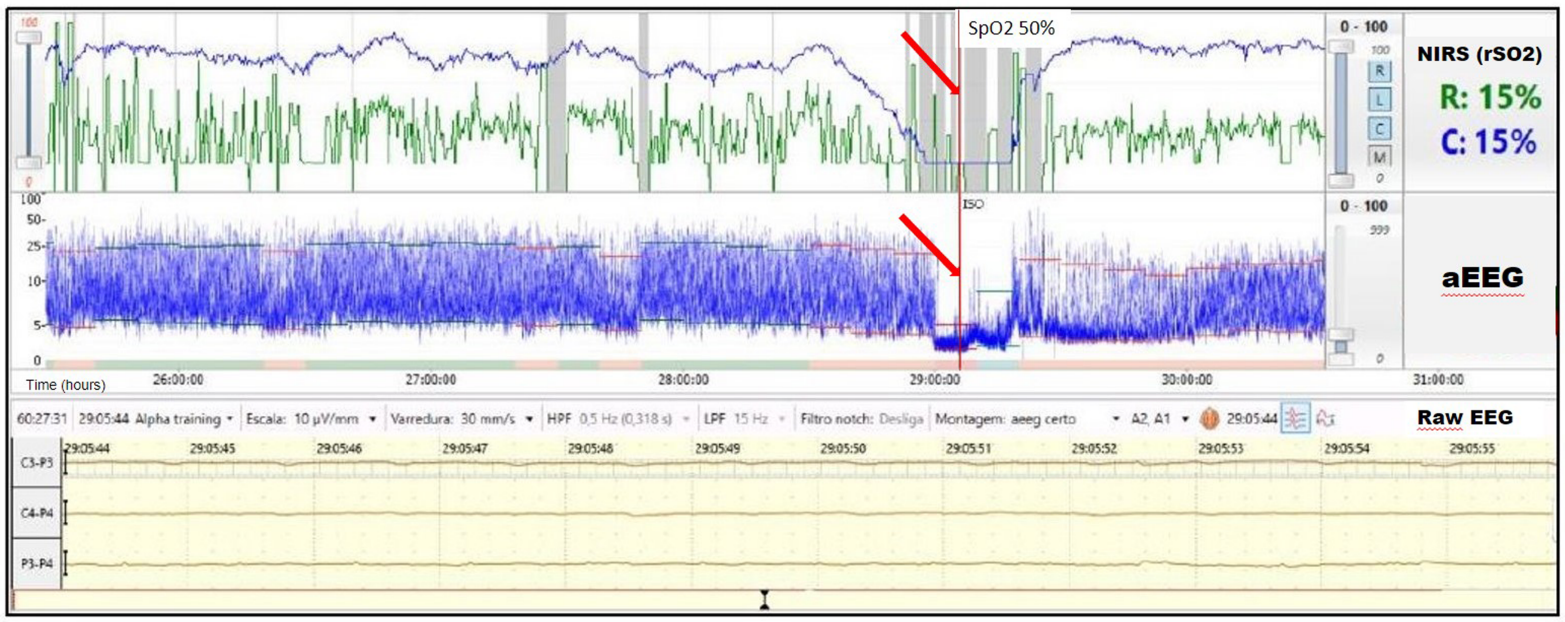
A 10-min decrease in cerebral saturation to 15% (blue trend line) was accompanied by a flat trace noted on aEEG (arrow) and raw EEG, followed by immediate aEEG background activity recovery after the increase of rScO_2_.

**Figure 6:**
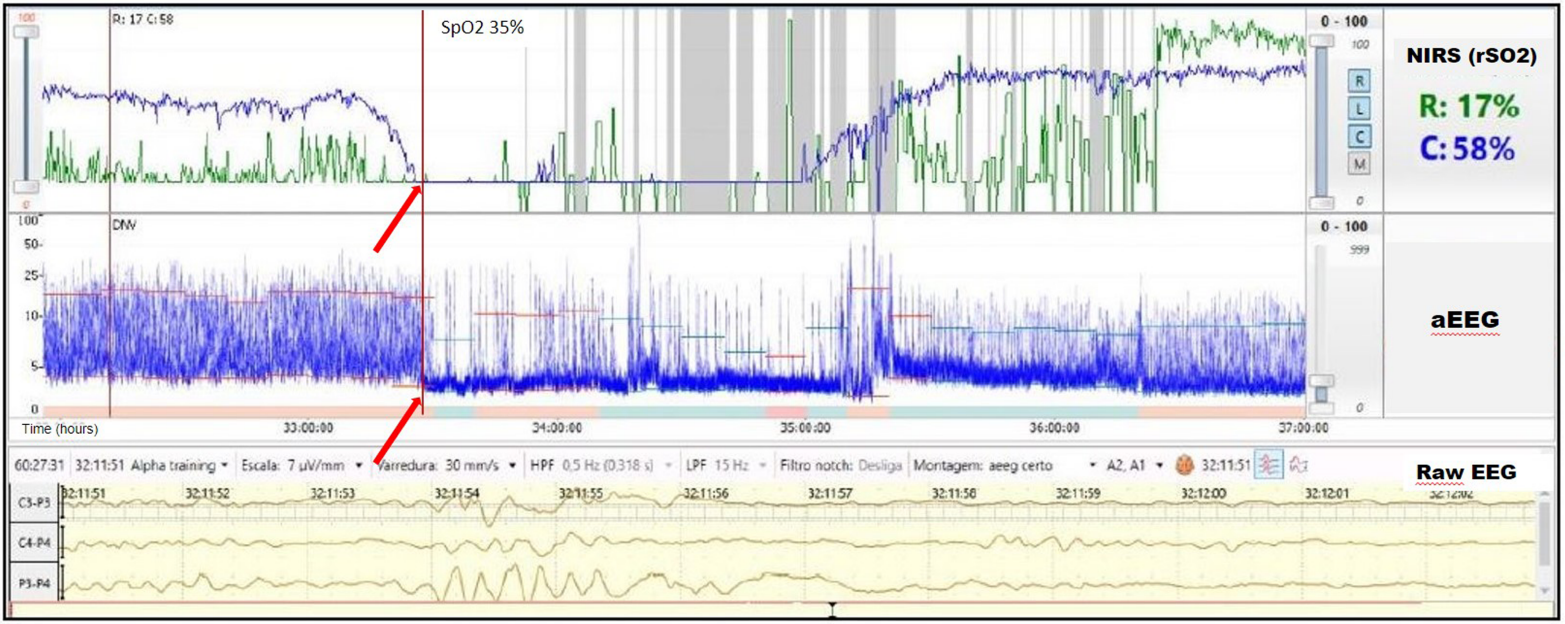
Prolonged arterial and cerebral desaturation with SpO_2_ ∼35% and rScO_2_ 15% (blue trend line) for 90 min associated with delayed recovery of aEEG background activity (arrows).

A term infant diagnosed with meconium aspiration and mild HIE developed severe and refractory pulmonary hypertension on day of life 1, requiring intubation and the use of inhaled nitric oxide. On day of life 2, an echocardiogram was performed and showed a hemodynamically significant PDA (hsPDA) measuring 2.9 mm. The infant had a prolonged desaturation to SpO_2_ 50%, followed by a decrease in cerebral saturation to 15%. These NIRS changes were accompanied by a flat trace on aEEG (Figure 5). Increased ventilator settings and FiO_2_ resulted in an increase in SpO_2_ and cerebral saturation recovery to a rScO_2_ ∼80%. This was quickly followed by an improvement in aEEG background pattern to discontinuous normal voltage. On the next day, the infant experienced a prolonged arterial and cerebral desaturation to SpO_2_ ∼35% and rScO_2_ 15% for a 90-minute period (Figure 6). Even after rScO_2_ recovery, the aEEG background activity remained severely abnormal for several hours. In this case, the cumulative effect of a prolonged desaturation was evident with persistently abnormal brain function.

#### Physiology

Due to a low metabolic rate for oxygen, healthy neonates demonstrate considerable reserve capacity to deal with reduction of cerebral blood flow or decrease of oxygen content in arterial blood (19). However, in previous studies, prolonged hypoxia (SaO_2_ ≤ 84%) for more than 30 s was associated with a significant reduction in systemic and regional oxygenation (cerebral and renal) in preterm infants. The intensity and prolongation of desaturation may compromise tissue oxygen utilization, leading to the risk of ischemic injury to the brain and kidney (14). Alderliesten et al. (20) conducted a study with 66 preterm infants ≤32 weeks of gestational age without a patent ductus arteriosus and treated for hypotension using inotropes. The authors found that neonates with a cerebral oxygen saturation lower than 50%, measured using small-adult sensors, for more than 10% of the time during the first 72 h after birth had worse neurodevelopmental outcomes at 18 months of age. It is important to consider that previous studies, mostly conducted in experimental settings, suggest that the neonatal sensor linearly correlates and reads ∼10% higher than the adult sensor. This information must be considered when analyzing cut-offs values for hypoxia and hyperoxia (21–23). The combination of low rScO_2_ and abnormal electrocortical activity likely reflects decreased cerebral oxygen delivery or cerebral blood flow and should alert the clinician to the risk for brain injury. Several investigators studied the relationship between electrocerebral activity measured by aEEG and measures of tissue oxygen delivery and consumption measured by NIRS. Ter Horst et al. (24) investigated the association between rScO_2_, cFTOE, and aEEG in 46 premature babies. The aEEG background activity had increasing continuity and there was higher cFTOE with advancing postnatal age. The authors concluded that there is an association between electrocortical activity and oxygen consumption. Our group recently described the combined use of both monitoring techniques in a wide range of clinical scenarios in which abnormal NIRS values were associated with alteration in electrocortical brain activity (6). The presence of a hsPDA together with cerebral autoregulation may explain the lower and highly variable renal saturation values during most of monitoring period. The left-to-right shunt is associated with increased pulmonary blood flow and decreased systemic blood flow, which may adversely affect both perfusion and oxygenation to the brain and kidneys. Neonates with a hsPDA may have lower cerebral and renal saturations. On the other hand, cerebral autoregulation may allow adequate blood flow to the brain despite this left-to-right shunt, while the kidneys will be more commonly affected. Chock et al. (25) previously conducted a study in preterm population, showing that lower renal saturation (rSrO_2_ < 66%) was related with the presence of a hsPDA.

### Case 6—Anemia

#### Case summary

**Figure 7:**
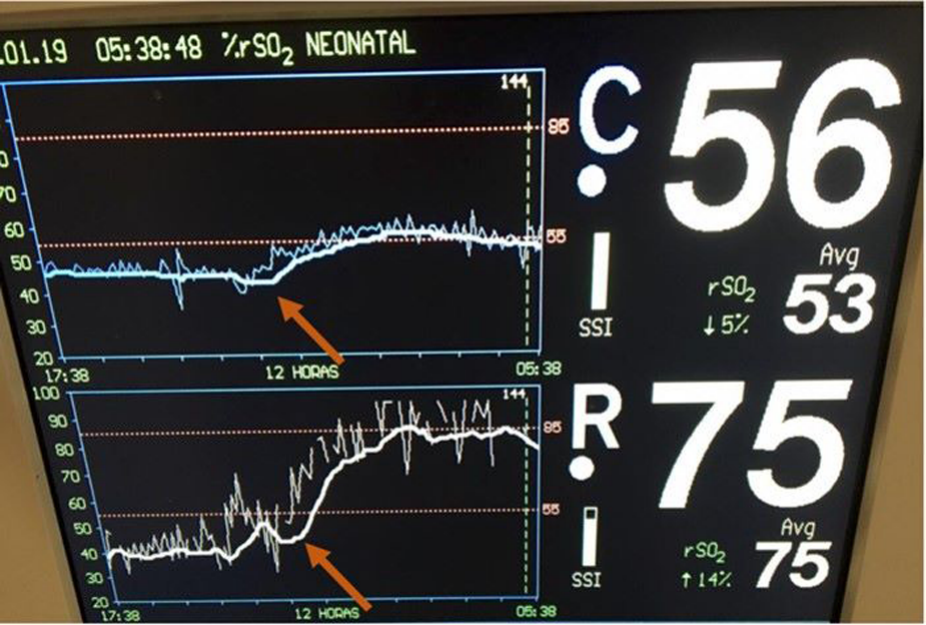
Low cerebral and renal saturations noted in NIRS monitoring with an immediate increase in rScO_2_ to ∼55% (blue trend line) and rSrO_2_ to ∼75% to 85% (white trend line) following PRBC transfusion (arrows).

An extremely preterm infant born by cesarean section with a birth weight of less than 1,000 grams, required intubation in the delivery room due to apnea. The infant was extubated at seven days of life to continuous positive airway pressure (CPAP), but on the second week of life was diagnosed with late-onset sepsis with positive blood culture for oxacillin-resistant coagulase-negative staphylococcus. Lumbar puncture excluded associated meningitis. NIRS monitoring was initiated, and rScO_2_ was ∼45% and rSrO_2_ ∼40%. Hemodynamic parameters were evaluated and blood pressure, peripheral perfusion, heart rate, systemic saturation and urine output all were within the normal range. Laboratory exams were performed and included PaCO_2_ 37 mmHg, Hemoglobin 10.6 g/dl and Hematocrit 30.2%. The infant was transfused PRBCs with an increase in rScO_2_ to ∼55% and rSrO_2_ to ∼75% to 85% (Figure 7). A 10-point decrease in heart rate (from 155 to 145 bpm) was noticed while the other hemodynamic parameters remained in the normal range.

#### Physiology

Hemoglobin levels directly influence tissue oxygenation, and low hemoglobin levels will have a direct impact on both regional cerebral and somatic saturation. Significant changes in oxygen supply may not be identified by pulse oximetry or other vital signs but may be detected at an earlier time point by monitoring regional saturation. When anemia is treated, both brain and somatic oxygenation improve. Whitehead et al. (26) studied a cohort of 68 infants born ≤ 30 weeks of gestational age and prospectively collected rScO_2_ values from the second week of life through 36 weeks postmenstrual age. Worsening anemia was associated with a progressive decrease in rScO_2_ in preterm infants. The identified threshold hemoglobin was 9.5 g/dl below which rScO_2_ values decreased >2 SD below the mean. Two recent systematic reviews using NIRS to detect anemia reported a significant correlation between hemoglobin concentration and rScO_2_ values, even when hemoglobin level was within the normal range (27, 28). The results suggest a reduction in the number of red blood cell transfusions per infant when NIRS was used to guide the transfusion decisions. The use of cerebral tissue oxygen saturation to guide individualized red blood cell transfusion therapy in the NICU is plausible and warrants additional exploration with the goal of improving both short- and long-term neurodevelopment in preterm children.

### Case 7—vein of galen malformation

#### Case summary

**Figure 8:**
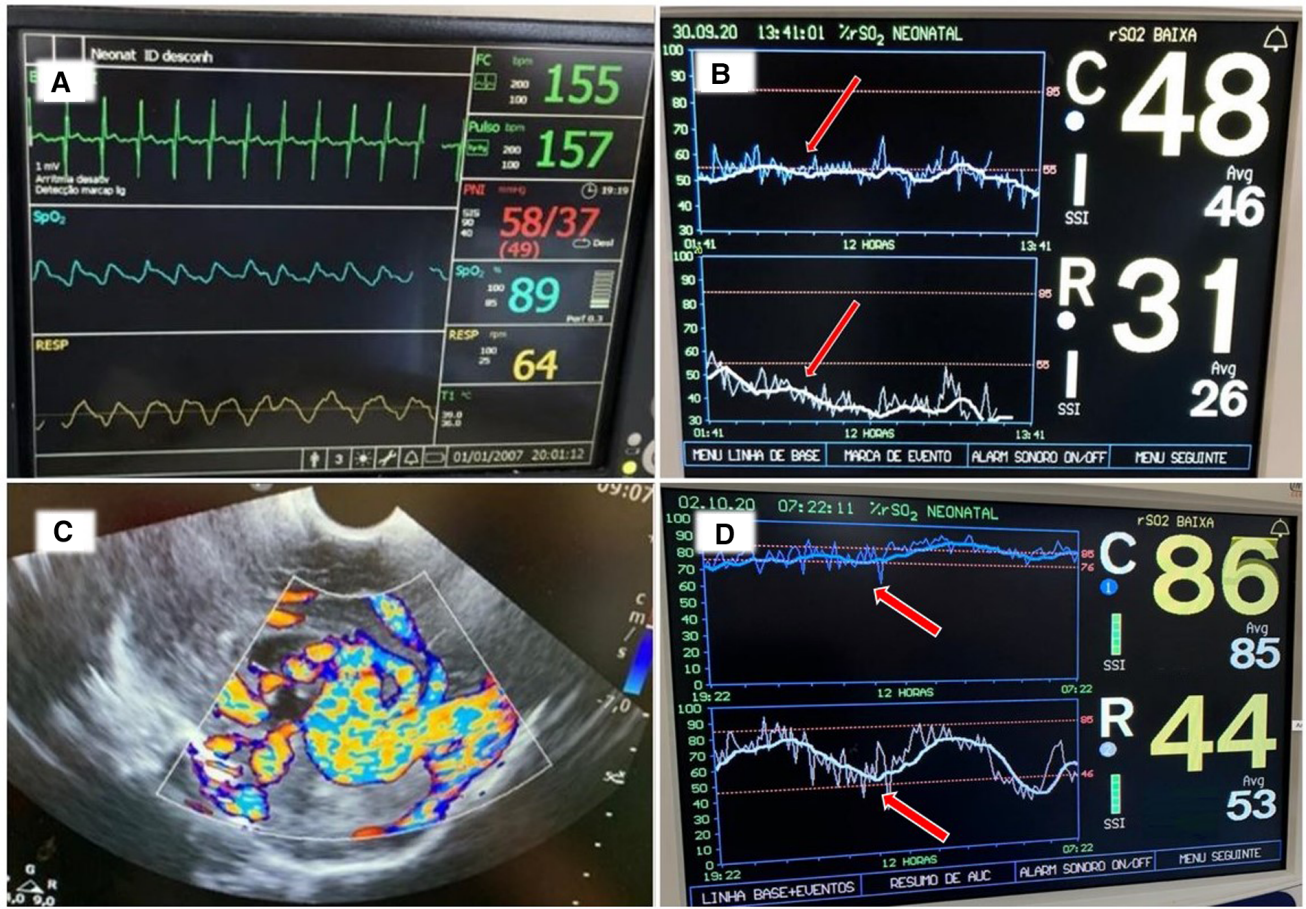
(**A**) Vital signs monitor with values within the normal range. (**B**) NIRS monitoring on the second day of life shows rScO_2_ (blue trend line) ranging 46%–50% while rSrO_2_ (white trend line) progressively declined with marked variability seen in both sites (arrows). (**C**) Cranial ultrasound showed an anechoic area located in the midline and associated with high flow compatible with vein of Galen aneurysmal malformation. (**D**) NIRS monitoring on day of life 5 shows an increase in both rScO_2_ (blue line) and also in rSrO_2_ (white line), but with marked variability in rSrO_2_. The infant received a PRBC transfusion due to a Hct 27% (arrows).

A late preterm infant was born by cesarean section and admitted to the NICU with mild respiratory distress. On the 2nd day of life, the infant required inotropic support for poor peripheral perfusion and oliguria. Heart rate, blood pressure and systemic saturation were within the normal range (Figure 8A). Blood gas showed pH 7.28, PaCO_2_ 34 mmHg, bicarbonate 16 mEq/l, and lactate level was 2.8 mmol/L. The rScO_2_ values were ∼46% to 50%, and a progressive decrease in rSrO_2_ to minimum of 26%, in addition to marked variability in both sites was noted (Figure 8B). On the second day of life a cranial ultrasound was consistent with a vein of Galen aneurysmal malformation (Figure 8C). The infant received cardiac catheterization with partial embolization of the malformation on the fourth day of life. An increase in both rScO_2_ and rSrO_2_ was noted, but with marked variability in rSrO_2_ (Figure 8D). During this period the infant presented with frequent, short periods of desaturation to SpO_2_ ∼80%. The remaining vital signs (heart rate, blood pressure and temperature) and blood gas findings were normal. The infant received a PRBC transfusion due to a Hct of 27%. Sepsis screening was done, and a coagulase-negative staphylococcal infection was diagnosed and treated with antibiotics.

#### Physiology

The vein of Galen aneurysm is a malformation with multiple markedly dilated arteriovenous fistulas that can divert up to 60% of cardiac output, leading to heart failure. The diagnosis can be made through imaging in the fetal or neonatal period, and cerebral angiography is the gold standard to confirm the diagnosis (29). NIRS provides information that may assist in the diagnosis and hemodynamic management of these infants. NIRS assists by reflecting whether cardiac output is sufficient to maintain adequate somatic and cerebral perfusion. Pichler et al. (12) evaluated whether simultaneous cerebral and peripheral NIRS monitoring together with a dedicated intervention guideline could avoid arterial hypotension and catecholamine administration in a cohort of 49 preterm infants. The burden of hypotension was reduced when NIRS monitoring was used to guide the management of hypotension. Due to redistribution of blood flow occurring with hemodynamic compromise, the early influence on somatic tissue oxygenation has also been demonstrated in neonates with congenital heart disease (13) and in children with dehydration needing volume resuscitation (30). A recent systematic review evaluated 41 studies of peripheral muscle NIRS measurements in clinical care of term and preterm neonates. The authors concluded that peripheral muscle NIRS data, either alone or in conjunction with cerebral or multi-site NIRS measurements, provides useful supplementary information concerning peripheral perfusion and oxygenation in critically ill newborns. This approach is a promising tool for detecting the initial stages of compensated shock in this vulnerable population (31).

## Abdominal disorders

### Case 8—gastroschisis

#### Case summary

**Figure 9:**
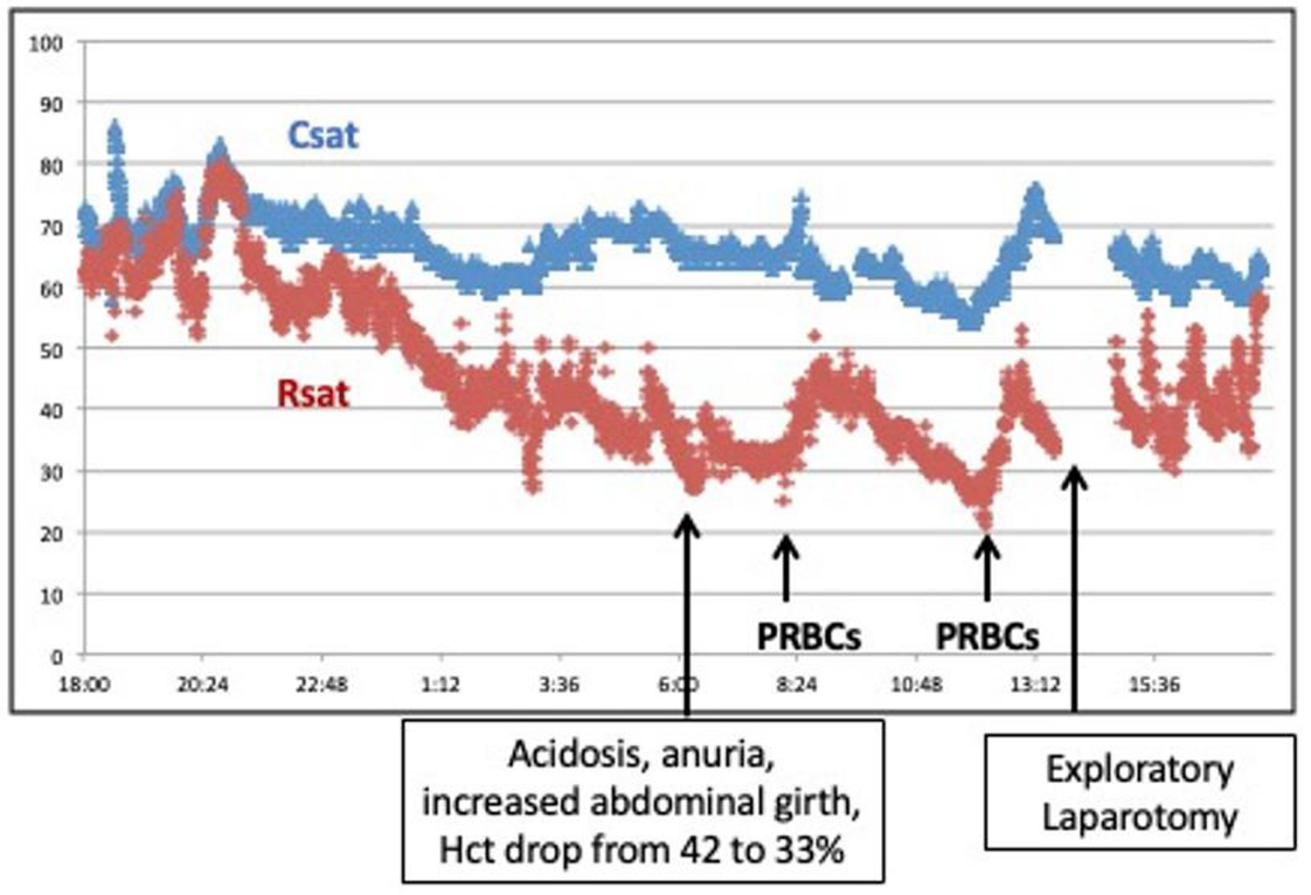
A significant decrease in renal saturation in an infant with gastroschisis was accompanied by progressive acidosis, anuria, increasing abdominal girth, and anemia. NIRS monitoring changes contributed to an early diagnosis of abdominal compartment syndrome.

A term infant with prenatal diagnosis of gastroschisis was admitted to the NICU with multiple loops of bowel outside of the abdomen. After elective intubation and sedation, the pediatric surgery team placed the bowel into a silo bag for gradual reduction. NIRS monitoring of cerebral and renal saturation was initiated following the surgical procedure. Initially the rSrO_2_ was trending very closely with rScO_2_ measuring ∼70% to 80%, but on day 3 of life over an 8-hour period there was a steady decrease in the rSrO_2_ to 30%–40% (Figure 9). The SpO_2_ remained 96%–99% and mean arterial blood pressure was stable at 37–45 mm Hg. An increase in abdominal girth and anuria was noted. The infant's hematocrit (Hct) decreased from 42% to 33% over a 24-hour period, and he developed a metabolic acidosis. He was transfused with 10 ml/kg of packed red blood cells (PRBCs) with a transient increase in his rSrO_2_ to 50%, but his rSrO_2_ decreased further to 20%–30%. The persistent decrease in renal saturation despite multiple transfusions prompted an exploratory laparotomy. The infant was found to have a subcapsular liver hematoma causing abdominal compartment syndrome. After bleeding was controlled and the bowel exteriorized to relieve pressure, the rSrO_2_ stabilized at 60%.

#### Physiology

Infants with gastroschisis undergoing active bowel reduction are at risk for abdominal compartment syndrome and bowel ischemia. NIRS monitoring during this time period, with particular attention to renal saturations as a surrogate for mesenteric perfusion, may be beneficial for early detection of gut ischemia. Renal and mesenteric blood flow are less well autoregulated compared to the brain, and in the setting of stable pCO_2_ levels, the cerebral saturation in this case was maintained for a longer time period. However, anemia from blood loss combined with abdominal compartment syndrome impacting perfusion to both intestines and kidneys likely contributed to the significant decrease seen in renal saturation. Stienstra et al. (32) reported application of the NIRS sensor directly onto the silo containing bowel to measure saturation levels. In their small pilot study of 12 neonates with gastroschisis undergoing staged closure, saturations were lower in those with confirmed bowel ischemia. A similar case report of an infant with gastroschisis demonstrated that NIRS monitoring of intestinal oxygen saturation decreased and corresponded to notable discoloration of a loop of bowel at the base of the silo. These findings reversed with surgical intervention (33). Continuous NIRS monitoring of intestinal or renal oxygenation is a useful modality to non-invasively assess changes in abdominal domain perfusion for infants with gastroschisis during the critical phase of bowel reduction.

### Case 9—seizures

#### Case summary

**Figure 10:**
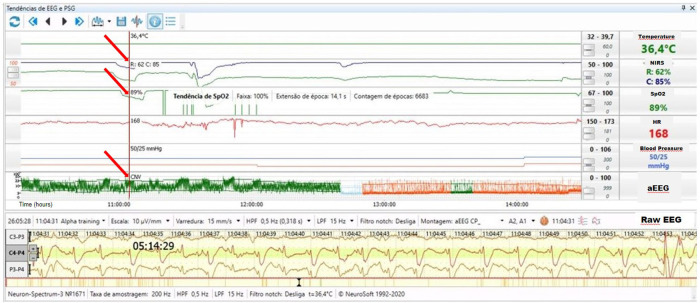
Electrographic seizure activity on raw EEG accompanied by an increase in heart rate to 168 bpm, slight reduction in systemic saturation to 89%, decreased rScO_2_ (blue trend line) from 95% to 85% and rSrO_2_ (green trend line) from 75% to 62% (arrows).

A term infant was born by cesarean section due to gastroschisis. The pediatric surgery service placed the bowel into a silo bag after birth for gradual reduction. On the 3rd day of life, the infant received sedation, analgesia, and neuromuscular blockade to facilitate the reduction. He developed electrographic seizures accompanied by an increase in heart rate to 168bpm and reduction in systemic saturation from 97 to 89%. The rScO_2_ decreased from 95% to 85% and rSrO_2_ from 75% to 62% (Figure 10).

#### Physiology

The incidence of seizures is high in newborns due to the relative excitability of the neonatal brain and high-risk of acute cerebral injury (34). Several investigations using EEG have demonstrated that most neonatal seizures have no clinical manifestations (electrographic only seizures), and that clinical skills alone are unable to distinguish epileptic seizures from non-epileptic events (35). Wallois et al. (36) reported the benefits of using NIRS and EEG together for continuous neuromonitoring, providing information regarding hemodynamic changes during seizures. In case 9, the high rScO_2_ may be explained due to low oxygen extraction from the brain due to sedation, analgesia and possible brain injury. The seizures were associated with a decrease in NIRS and SpO_2_ values and may also be an indicator of brain injury. Although the abnormal NIRS values could be a consequence of the decrease in SpO_2_, information about end organ oxygenation remains valuable for clinical management. Given that NIRS readings reflect the balance of oxygen delivery and regional consumption, the higher cerebral metabolic demand could explain a drop in rScO_2_ during seizures. The level of cerebral deoxygenation during seizures may be used to anticipate risk, assess the impact of treatment, and even inform new seizure classification in the future.

### Case 10—NEC and feeding intolerance

#### Case summary

**Figure 11:**
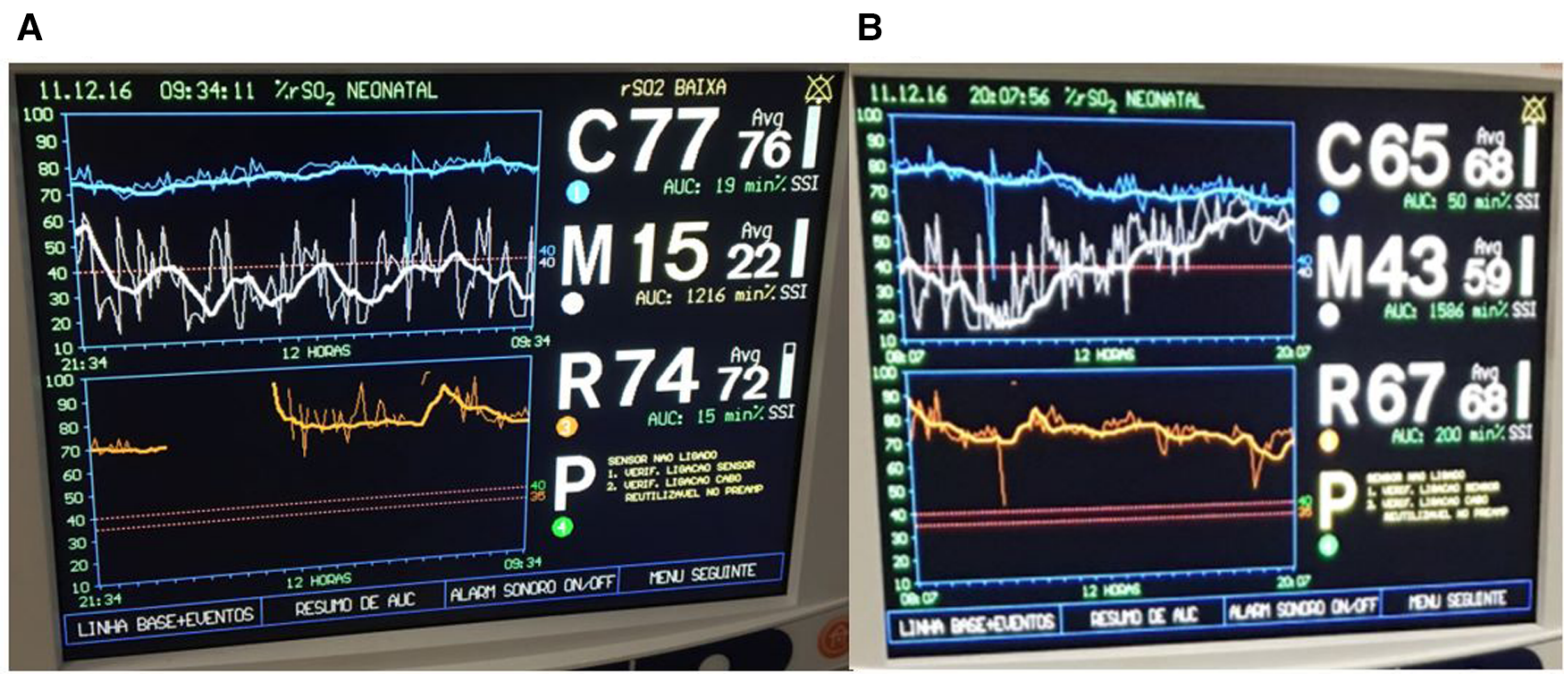
(**A**) Decline in mesenteric saturation (white trend line) with increased variability noted after a red blood cell transfusion. (**B**) After diagnosis of necrotizing enterocolitis, infant was intubated and started on antibiotics with improvement in mesenteric saturation.

A small for gestational age (SGA) moderately preterm infant was having frequent apnea and bradycardic episodes in the third week of life. CPAP support was initiated. Hematocrit was 25% and PRBCs were transfused. Over the next 12-hour period, the infant developed significant abdominal distention. NIRS tracing demonstrated a notable decrease in mesenteric saturation from the 55%–65% range down to 15%–20% (Figure 11A). An abdominal radiograph showed signs of pneumatosis intestinalis. After this diagnosis of NEC, the infant was subsequently made NPO, started on antibiotics, and intubated with a slow recovery in mesenteric saturation (Figure 11B).

#### Physiology

NIRS has been investigated as a diagnostic tool in preterm infants at risk for spontaneous intestinal perforation and NEC. These gastrointestinal morbidities often present with non-specific clinical signs including apnea, abdominal distention, increased gastric residuals, or poor perfusion. Placement of a NIRS probe in the infraumbilical region to measure abdominal oxygenation may provide a marker of intestinal hypoperfusion. In this way, abdominal NIRS (also described as splanchnic or mesenteric) may help to identify infants with NEC earlier or provide reassurance during episodes of feeding intolerance in a preterm infant. Several authors have reported a decrease in abdominal rSO_2_ measures in preterm infants with subsequent feeding intolerance or NEC (37–41). Increased ratio of splanchnic to cerebral oxygenation (SCOR) (42) and lower cerebral saturation (43) has also been associated with NEC, while a decrease in SCOR may be seen with more severe NEC requiring surgical intervention (41). Others have found no difference in abdominal rSO_2_ or SCOR in preterm infants with and without NEC after onset of clinical symptoms (44, 45). Discrepancies in studies may be related to the unclear effect of peristalsis or underlying air or meconium on abdominal NIRS values. While NIRS monitoring of infants at risk for NEC may be useful, threshold values to identify NEC have yet to be established.

## Discussion

These ten neonatal neuromonitoring cases demonstrate new uses of NIRS and aEEG in the neonatal population and how these neuromonitoring techniques can provide valuable information regarding hemodynamics as well as brain function. While the use of NIRS in specific clinical scenarios has been well documented for several neonatal diagnoses such as HIE, congenital heart disease, and PDA (4, 13, 25, 46–52), there is less written about its use for other diagnoses encountered in the NICU. The cases described provide a clear rationale for the use of NIRS in several clinical scenarios; judging the need and response to blood transfusion, detection of bowel ischemia with bowel reduction for gastroschisis, determination of adequate cardiac output to maintain cerebral and somatic perfusion with vein of Galen malformation, recognition of hypercapnia-induced cerebral hyperperfusion due to pneumothorax, low cerebral and renal NIRS caused by hyperkalemia induced bradycardia and hypotension, use of two-site NIRS to identify a silent pericardial effusion on ECLS, and use of abdominal NIRS in setting of feeding intolerance and NEC. Two-site NIRS and aEEG were used together in three cases in our series. In the first case, a newborn with PPHN experienced a short cerebral desaturation episode with no effect on cerebral function as measured by aEEG and then a later more prolonged episode of cerebral desaturation with a profound effect on brain function. The second case was a neonate with severe left diaphragmatic hernia where NIRS and aEEG were used together to guide management and the decision regarding ECMO cannulation. The third case was a term infant with gastroschisis and electrographic seizures accompanied by tachycardia as well as a transient decrease in cerebral and renal saturation likely due to increased metabolic demand. These three cases highlight the benefits of multi-modality monitoring enabling providers to understand changes in hemodynamics and oxygenation of the brain and somatic tissues as well as the effect on cerebral function. NIRS monitoring has several strengths; it allows for non-invasive real-time monitoring of tissue oxygenation at the bedside, is an early biomarker of abnormal tissue oxygenation; and can be applied in a variety of settings, including the intensive care unit, during transport or surgeries. However, NIRS findings need to be interpreted in the context of the underlying physiology of each monitored site. Physiological variables such as oxygen content, circulation, and oxygen extraction directly influence rSO_2_ values (53). Limitations of NIRS monitoring also include limited depth of near-infrared light penetration and unclear organ specificity, especially in somatic NIRS monitoring which may reflect oxygenation of surrounding tissues. Also, NIRS readings may be affected by external factors such as light, motion and temperature (54). The main strengths of aEEG include non-invasive, safe, and real-time monitoring of brain function, yet being limited by a reduced number of channels and the need for training providers in aEEG interpretation (55).

## Conclusion

With understanding of the underlying principles of NIRS as well as the physiologic factors which impact oxygenation and perfusion of end organs, changes in neonatal physiology can be more easily recognized by bedside providers and diagnostic and therapeutic interventions can be undertaken. aEEG used in conjunction with NIRS provides an enhanced understanding of brain function in settings where brain injury is suspected. Normal background patterns are reassuring while abnormal background patterns confirm concerns regarding abnormal brain function. Considering the clinical benefit of better understanding physiology in critically ill neonates, we anticipate that there are a myriad of uses of NIRS and multimodality monitoring in populations at risk of hemodynamic disturbances which will continue to be reported.

## Data Availability

The original contributions presented in the study are included in the article, further inquiries can be directed to the corresponding author.
